# Molecular characterization of the first reported *Aichivirus* A in Australia

**DOI:** 10.1099/acmi.0.000099

**Published:** 2020-02-11

**Authors:** Judith A. Northill, Russell J. Simmons, Doris Genge, Frederick A. Moore

**Affiliations:** ^1^​ Public Health Virology, Forensic and Scientific Services, Coopers Plains, QLD, Australia

**Keywords:** *Aichivirus*, real-time RT-PCR, gastroenteritis

## Abstract

A novel real-time reverse transcription polymerase chain reaction (RT-rPCR) assay was developed to detect *Aichivirus A* (AiV-A) based on four complete genomes. The assay successfully detected AiV-A in a sample from a patient with acute gastroenteritis in January 2008. Screening of 756 samples submitted for norovirus testing during May 2008 detected a further 23 AiV-A-positive samples from 18 individual patients. Genotyping using novel primers targeting the 3C–3D junction region identified AiV-A genotype B. Further sequencing of the VP1 region supported the 3C–3D result. All three assays proved useful to support foodborne outbreak investigations. This is the first report of AiV-A detection in Australia.

## Introduction

*Aichivirus A* (AiV-A) viruses were first detected in samples from an oyster-related gastroenteritis outbreak in Aichi, Japan in 1989 [1]. AiV-A viruses are species of the family *Picornaviridae* sharing the genus *Kobuvirus* with five other species identified as *Aichivirus B*–*F* [2]. AiV-A are a group of single-stranded, positive-sense RNA viruses that cluster into two confirmed genotypes, A and B [3], with a third genotype proposed [4].

The first complete AiV-A genome was determined in 1991 [5]. AiV-A has since been identified in many countries including, Bangladesh, Thailand, Vietnam [6], Germany, Brazil [7], France [4], Tunisia [8], Hungary [9], PR China [10], Finland [11], Spain [12], India [13] and Sweden [14]. These global viruses have also been reported in polluted water in Venezuela [15] and sewage in Tunisia [16].

Here we report the first detection of AiV-A in Australia. We detail the development of a novel screening RT-rPCR assay and two new conventional agarose gel-based RT-PCRs for genotyping in the 3C–3D and VP1 regions. We discuss the amino acid differences between genotypes within the capsid sequences that support previous reports [6] and report additional sequences of AiV-A that further aid assay development or improvements for the identification of foodborne gastroenteritis clusters.

## Methods

### Specimens

The initial AiV-A detected sample type was a faecal sample from a female patient. The further study was conducted on 756 samples. The majority were faecal samples (739), while the remaining 17 were vomitus.

### Nucleic acid extraction

Viral RNA was extracted from a 10 % faecal suspension using the Qiagen BioRobot Universal System and the QIAamp Virus BioRobot MDx kit (Qiagen, Australia).

### Real-time RT-PCR for detection of AiV-A RNA (AiV-A RT-rPCR)

A novel RT-rPCR was designed for AiV-A (Table 1) using Primer Express software (Life Technologies, USA). The 106 nt target region was located in the AiV-A 3′ UTR (GenBank accession number DQ028632) and the chosen probe and primers were aligned and compared to other sequence information available in 2007. Assay-specific synthetic templates (Geneworks Pty Ltd, Australia) were devised as a long-term consistent control. Details of the method to produce these binary controls are outlined in a previous publication [17]. Both controls were prepared and titrated for use (data not shown). A cycle threshold (*C*_T_) of <40 indicated a positive result with a *C*_T_>40 indicating that the template was not detected. The reaction mix was formulated using the Invitrogen SuperScript III Platinum One-Step Quantitative RT-PCR System (Life Technologies, Australia), 300 nM of both forward and reverse primer and 150 nM of probe, and 5 µl of RNA extract or diluted synthetic control was added for a final volume of 20 µl [18]. RT-rPCR was performed using a Corbett Rotor-Gene 6000 (Qiagen, Australia) with cycling conditions described elsewhere [18].

**Table 1.** Diagnostic and genotyping oligonucleotides

Assay name	Oligonucleotide name	Sequence (5′−3′)	*^a^*Location
AiV-A RT-rPCR^*b*,*c*^	AiV-F-8046	TGCTTCGGCACGCTTAGTT	8046–8064 (3′ UTR)
	AiV-R-8151	TGCARTACAACCAYGGCTTAGG	8120–8151 (3′ UTR)
	AiV-8082-FAM	6FAM-CACTCCTCCATGGTGATATAAAGACCAC-TAMRA	8082–8109 (3′ UTR)
3C–3D RT-PCR^*d*^	AiV-6213F	ACTGGGCCACCCTCCAGACG	6228–6247 (3C)
	AiV-7044R	GGTTGATTTCAGCTTGGAGTTC	7058–7037 (3D)
VP1 RT-PCR^*d*^	AiV-2789F	TTCACCATCCCCTTCATCTC	2804–2823 (VP3)
	AiV-4302R	GCAAGGGAGACAGAATTTGC	4317–4298 (2B)
	AiV-3867R	GGGGAGACCTTGCGGATRGC	3882–3863 (2A)
Yamashita 3C-3D RT-PCR^*e*^	AICHI-6261	ACACTCCCACCTCCCGCCAGTA	6276–6297 (3C)
	AICHI-6779	GGAAGAGCTGGGTGTCAAGA	6794–6775 (3D)

*a,* Location is based on the GenBank AiV-A strain, accession number DQ028632.

*b,* Designed in 2007 using an alignment of available sequences, AB010145, AB040749, AY747174 and DQ028632.

*c,* Designed using Primer Express software (Life Technologies, USA).

*d,* Designed using Primer3 (http://primer3.ut.ee/).

*e,* Published by Yamashita *et al*. [3].

### Conventional RT-PCRs for initial subgenomic sequencing and full-genome Sanger sequencing

A conventional RT-PCR from a published set of primers (Table 1) was used to generate a 519 bp product [3]. This RT-PCR is referred to as the ‘Yamashita 3C–3D conventional RT-PCR’. The reaction mix was formulated using the Invitrogen SuperScript III One-Step RT-PCR System with Platinum *Taq* High Fidelity (Life Technologies, Brisbane Australia). To this mix, 5 µl of extracted RNA was added for a total reaction volume of 20 µl. For mix details and cycling times see the detailed protocol published elsewhere [19]. This RT-PCR was used to confirm the first detected sample by AiV-A RT-rPCR, but it could not detect all the subsequent samples, so a new set of primers was designed for this region.

Further specific sets of oligonucleotides (available upon request) were designed using the complete genome of four AiV-A strains (AB010145, AB040749, AY747174 and DQ028632) as a guide. Multiple RT-PCRs were performed using 500 nM of primers and the Invitrogen Superscript III One-Step RT-PCR System with Platinum *Taq* kit. The cycling times were 55 °C for 15 min, 94 °C for 2 min, followed by 40 cycles of 94 °C for 15 s, 52 °C for 30 s and 68 °C for 20 s. Amplified products from these RT-PCRs were purified using the Qiagen QIAQuick PCR Purification kit (Qiagen, Australia) and sequencing was performed using the Big Dye Terminator Cycle Sequencing Ready Reaction kit version 3.1 (Applied Biosystems, Australia) and an ABI3130xl Genetic Analyser (Applied Biosystems, Australia). The genome ends were determined using the 5′/3′ RACE kit, second Generation (Roche, Germany).

### Conventional RT-PCRs for additional subgenomic sequencing

**Fig. 1.**
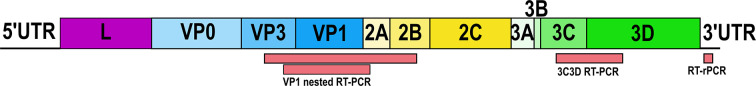
Schematic of AiV-A based on the sequence from GenBank AB040749. The target regions of the 3C–3D RT-PCR, VP1 RT-hnPCR and AiV RT-rPCR are underlined. UTR, untranslated region; L, non-structural leader protein; VP0, VP3 and VP1, structural viral proteins, P1; 2A, 2B and 2C,non-structural proteins, P2; 3A, 3B, 3C and 3D, non-structural proteins, P3.

Two new conventional RT-PCRs were designed for genotyping. Firstly, a newly designed RT-PCR targeting the AiV-A 3C–3D junction region (3C–3D RT-PCR) proved highly successful. The primers, AiV-6213F and AiV-7044R (Table 1), produced an 832 bp sequence that extends beyond most of the partial 3C–3D sequences publicly available in GenBank (Fig. 1). The reaction mix was formulated using the Invitrogen SuperScript III One-Step RT-PCR System with Platinum *Taq* High Fidelity (Life Technologies, Australia). To this mix, 5 µl of extracted RNA was added for a total reaction volume of 25 µl. For mix details and cycling times see the detailed protocol published elsewhere [20]. The 3C–3D RT-PCR was used to confirm all the samples detected by the RT-rPCR.

The second assay was a conventional hemi-nested RT-PCR targeting the VP1 region of AiV-A (VP1 RT-hnPCR). The VP1 is the most variable of the structural proteins [6] and ideally suited for genotyping purposes. This novel RT-PCR was designed using available sequences with the second round providing coverage of the full-length VP1 coding region. The RT-PCR reagents were prepared using the Invitrogen SuperScript III One-Step RT-PCR System with Platinum *Taq* High Fidelity kit (Life Technologies, Australia) and primers AiV-2789F and AiV-4302R (Table 1), with 5 µl of RNA added for a final reaction volume of 20 µl. A second-round PCR was amplified using the forward primer above, AiV-2789F, and AiV-3867R (Table 1). The reaction mix was formulated using the MyFi mix [Bioline (Aust) Pty Ltd, Australia] and 5 µl of the first-round PCR product diluted 1/100 for a total reaction volume of 20 µl. For mix details, primer concentration and cycling times see the detailed protocol published elsewhere [21]. The first-round PCR product was 1514 bp and the second round was 1079 bp in size.

### Nucleotide sequence analysis and phylogenetics

**Fig. 2.**
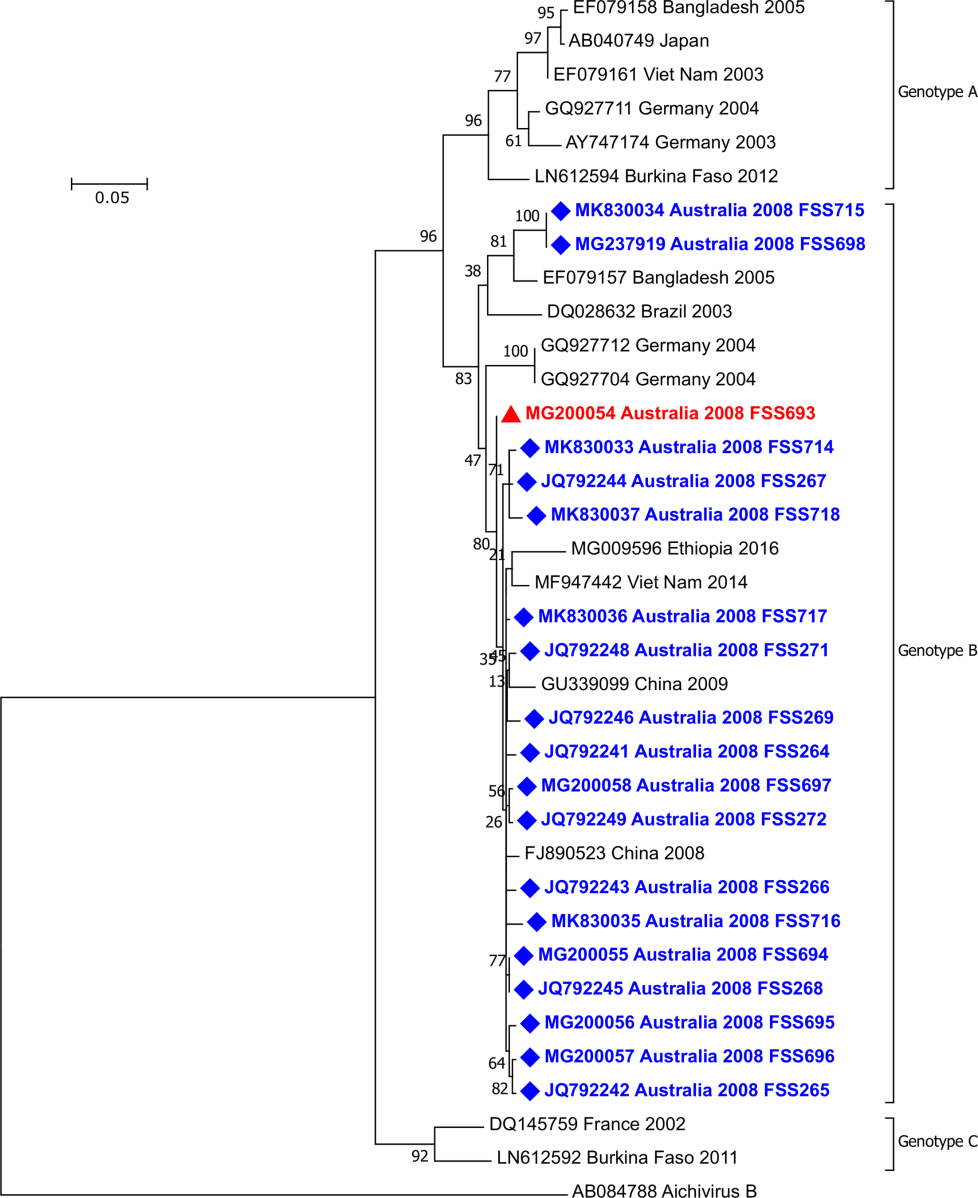
Phylogenetic analysis of 477 bp sequences in the 3C–3D junction region of AiV-A. Strains marked with a blue diamond relate to this study. MG200054 is the first detected Australian AiV-A (red triangle). JQ792240–JQ792249, MG200055–MG200058 and MK830033–MK830037 were the sequences detected in the May 2008 study. Note: a 477 bp fragment was analysed to accommodate the shorter genotype C GenBank sequences.

**Fig. 3.**
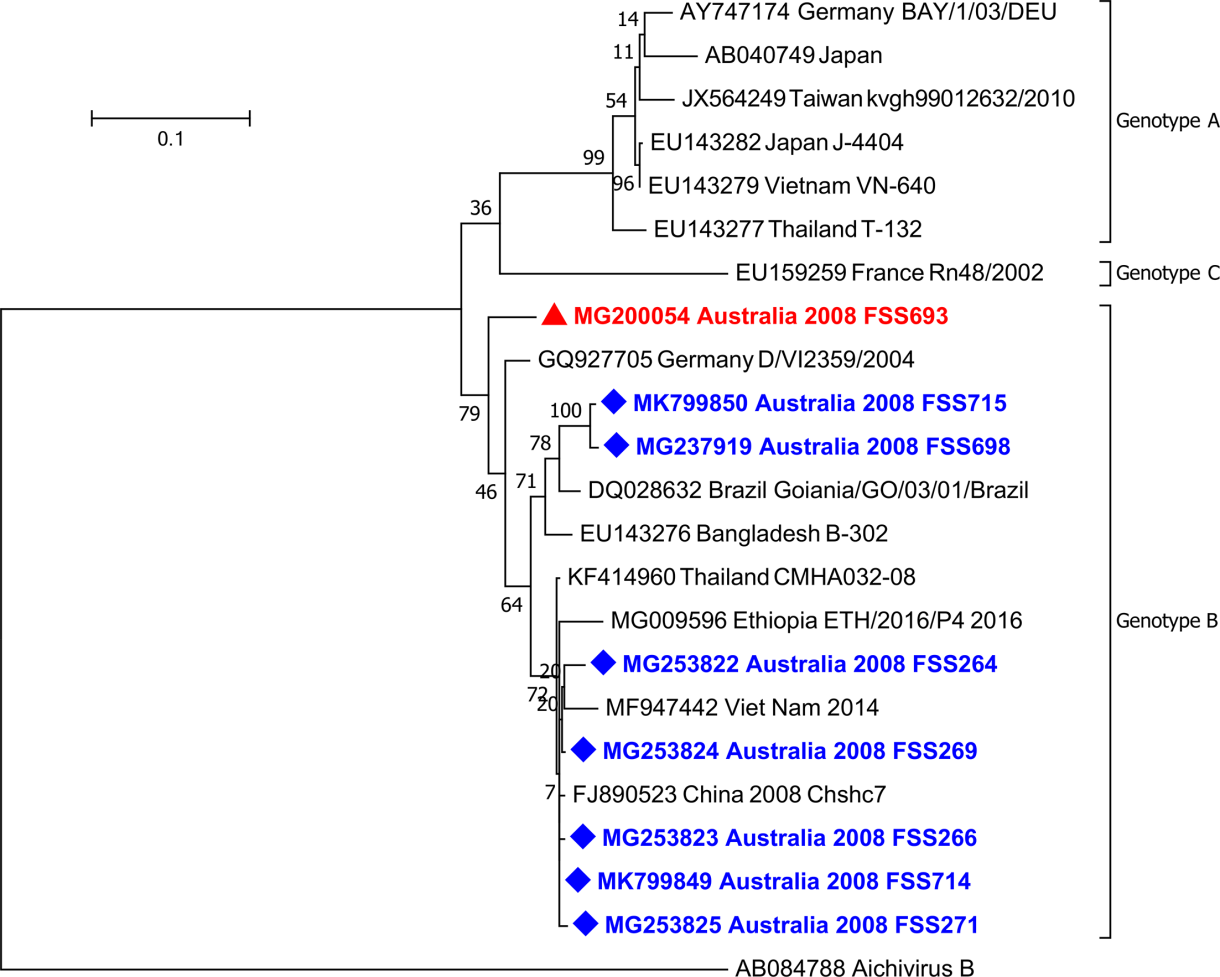
Phylogenetic analysis of 641 bp sequences in the VP1 region of AiV-A. Strains marked with a diamond relate to this study. MG200054 is the first detected Australian AiV-A (red triangle). Sequences MG237919, MG253822–5 and MK799849–MK799850 were the sequences detected in May 2008.

Overlapping sequences were assembled using Sequencher 5.0 (Gene Codes Corporation, USA) to construct a near full-length genome. Sequence alignment and phylogenetic analysis for both the 3C–3D and VP1 regions were performed using mega7 (22), removing the primer sequences. The 3C–3D sequences from the published RT-PCR (519 bp) and the new 3C-3C RT-PCR (832 bp) were analysed with GenBank sequences and trimmed to 477 nucleotides to accommodate the shorter genotype C sequences, DQ145759 and LN612592 (Fig. 2). The VP1 RT-hnPCR sequences (1079 bp) were aligned with shorter GenBank sequences and trimmed to 641 nucleotides for analysis (Fig. 3). Both phylogenetic trees were calculated using the maximum-likelihood method with 500 bootstrap replications using mega7 [22].

## Results

The new RT-rPCR assay successfully detected AiV-A reference material supplied by T. Yamashita, Aichi Prefectural Institute of Public Health, Japan. Synthetically produced controls, included in each run alongside no-template controls, were used to monitor ongoing assay performance during routine use of the assay. The RT-rPCR was added to a suite of assays employed to investigate suspected foodborne outbreaks of unknown origin.

### First detection of an AiV-A virus

In January 2008, a patient in Queensland presented with gastroenteritis of unknown aetiology. Testing of the RNA extract from a stool sample (FSS693) identified AiV-A at 29 cycles. The Yamashita 3C–3D RT-PCR was used to confirm the AiV-A RT-rPCR result. Sanger sequencing confirmed AiV-A and genotyping using the 519 bp sequence placed it in AiV-A genotype B (Fig. 2). This region of the genome is widely used [3] and the majority of partial GenBank sequences include this area. The nucleotide sequence was deposited in the GenBank database under accession number EU715251 and later updated with the longer sequence MG200054.

Further genomic sequencing of sample FSS693 was then conducted using sets of specific primers designed based on the complete genome of available AiV-A strains. Only the 5′ end of sample FSS693 could not be determined due to its difficult stem–loop structure [23]. Phylogenetic analysis of both the 3C–3D and capsid region showed that it belonged to genotype B and had an amino acid insertion of a proline in VP0 at position 223. The amino acid sites 219–223 varied throughout the available sequences, and FSS693 had an STNSP motif, the same amino acid sequence identified in three Bangladesh sequences, EU143274–EU143276. The full-length capsid region, VP0, VP3 and VP1, was analysed with additional capsid sequences from GenBank. The two groups differed at the nucleotide level by 11–14 %; FSS693 differed by 5–6 % compared to other genotype B sequences.

Isolation of FSS693 was also attempted by three blind passages in Vero cells, however it proved unsuccessful, with no CPE observed and an increase in *C*_T_s when each passage was tested using the AiV-A RT-rPCR, until it was not detected on the final passage.

### Further screening for *Aichivirus* A viruses

The detection of AiV-A in January 2008 prompted the investigation of additional faecal samples to gauge whether the virus was in circulation among in samples tested in the Queensland community. During May 2008, 756 faecal and vomit samples from 650 patients (55 % female) were submitted for norovirus testing and each of these samples was also screened using the AiV-A RT-rPCR as they arrived. The age distribution of patients is skewed to each end (Table 2).

**Table 2.** Age distribution of patients

Age group (years)	No.	Percentage of population
<1–4	154	23.7
5–19	44	6.8
20–39	47	7.2
40–59	75	11.5
60–99	330	50.8
Totals	650	100

There were 23 positive samples detected from a total of 18 individual patients (3 % of norovirus requests). The 18 patients ranged in age from 2 to 89 years, with 50 % of patients (*n*=9) aged 61–89 years old. The remaining patients were either children aged 2–18 years (*n*=6) or adults aged 19–60 years (*n*=3). Sample types included 1 vomitus and the remaining 22 were faecal samples. From the 18 patients with AiV-A, 7 were also co-infected with norovirus genotype 2. This is comparable to previous studies reporting viral co-infection, such as AiV-A with astrovirus, rotavirus or norovirus [4, 7, 24]. In one study two patients were co-infected with three viruses, AiV-A, rotavirus and astrovirus [24].

These samples were also genotyped using the 832 bp amplified product of the 3C–3D RT-PCR. All 18 patients’ viruses grouped within AiV-A genotype B; however, sequence variation was seen within the group of samples (Fig. 2). There were 2 sequences, MK830034 and MG237919, with a pairwise distance of 6 % compared to the remaining 16 sequences from this study. Sequences generated were deposited with GenBank.

### Analysis of Aichivirus A VP1

Eight of the Queensland May 2008 samples were tested using the VP1 RT-hnPCR. Seven were detected and able to be further sequenced. The sequences obtained were analysed alongside sequences from GenBank and a phylogenetic tree was created using mega7 (Fig. 3). A 641 bp length sequence region was aligned to accommodate the shorter GenBank sequences. All seven sequences were genotype B, which confirmed the 3C–3D result. The sequences fell into two distinct clades within genotype B, with two sequences having a pairwise nucleotide distance of 5–7 % compared to the remaining five sequences from this study. The sequences generated were deposited with GenBank.

## Discussion

Often there are undiagnosed cases of gastroenteritis and to address gaps in testing a screening assay for AiV-A was proposed. We designed an RT-rPCR for AiV-A in 2007 based on sequences available at the time. The AiV-A RT-rPCR allowed us to detect AiV-A in a faecal sample followed by identification of a further 18 cases from samples submitted for norovirus testing. This observational investigation of patients with AiV-A infection involved those aged from 2 to 89 years. While small, the findings revealed that AiV-A infects children and adults of all ages. Seroprevalence studies in Germany and Japan have shown that up to 80 % of adults have antibodies for AiV-A [7, 25]. Our study also revealed seven dual infections with noroviruses, with many previous studies reporting co-infection as commonplace, raising the question of whether AiV-A viruses are passengers or pathogens [4, 7, 24]. Recent studies have been unable to identify a causative role for AiV-A in gastroenteritis [26, 27], but longitudinal cohort studies are needed to better assess the role of AiV-As in acute gastroenteritis. Our first AiV-A-positive sample tested negative for *Norovirus*, *Mamastrovirus 1* and *Rotavirus A*, and conventional enteric bacteria. Eleven patients from the subsequent investigation had gastroenteritis of unknown aetiology, however; exhaustive testing for other viruses associated with gastroenteritis was not carried out, and further studies are warranted in Australia to determine the sustained ongoing burden of AiV-A in our population.

AiV-A viruses can be assigned to three genotypes, designated A, B and C [3, 4]. All 19 positive Queensland-detected viruses contained genotype B viruses according to sequences generated by our 3C–3D RT-PCR. Interestingly, the sequences demonstrated variability, falling into distinct clades within genotype B (Fig. 2). The 3C–3D RT-PCR may, therefore, be useful for contact tracing purposes in addition to its role in confirming the screening assay results. Genotype B viruses have been reported worldwide [7, 10, 13, 28]. From our 3C–3D phylogenetic tree (Fig. 2), Queensland viruses were seen to group together with sequences from predominantly East Asian and Southeast Asian countries.

The VP1 sequence is the most variable of the AiV-A regions [6], making it difficult to sequence but a valuable source of data for genotyping when sequencing is successful. Most recent sequences in GenBank are partial VP1 sequences, ending before a region of approximately 40 nucleotides with a high number of cytosines. Our study generated seven partial VP1 sequences, which were all genotype B, with five grouping together with sequences from China and Thailand and two most like sequences from Brazil and Bangladesh (Fig. 3). This supports the hypothesis that AiV-A is circulating throughout the community, possibly through multiple introductions over time, and not as a single strain or outbreak of the virus.

Further scrutiny of the VP0, VP3 and VP1 capsid regions of FSS693 revealed the formation of clades within both the genotype A and B lineages; however, the number of publicly available full-length capsid sequences is limited. The first four genotype A clades were reported in 2008 [6] when the full capsid gene was analysed, and additional full-length capsid sequences from Thailand, Taiwan, Viet Nam, Germany and China are now available in GenBank. There is still no full-length genotype C sequence, only short VP1 and 3C–3D sequences from Burkina Faso and France (Mali travel history [6]). The mean percentage nucleotide distance between the two genotypes for the capsid sequences is 12–14 %, with FSS693 having a mean percentage difference of 5–6 % to other genotype B sequences. The reported amino acid insertion [6] in the VP0 of genotype B sequences was also observed in the FSS693 sequence. Amino acid sites 219–223 vary among the sequences analysed, with FSS693 having the same STNSP motif as that seen in three Bangladeshi sequences.

To the best of our knowledge, these are the first AiV-As detected from acutely ill patients to be characterized in Australia. These novel assays support the detection of *Aichivirus A* when used to identify a pathogen in cases of gastroenteritis of unknown aetiology and their use highlights the need for more research on these rarely sought viruses. Our novel RT-rPCR diagnostic and conventional sequencing assays provided valuable information for understanding the identification, global spread and genetic variability of AiV-As circulating in Australia.
